# Is Digital Maxillary Model Scanning Reliable in Individuals with Unilateral Cleft Lip and Palate?

**DOI:** 10.3390/diagnostics15202553

**Published:** 2025-10-10

**Authors:** Elif Merve Mavi, Ozge Uslu-Akcam, Mehmet Okan Akcam

**Affiliations:** 1 Department of Orthodontics, Faculty of Dentistry, Ankara University, 06500 Ankara, Türkiye; 2 Department of Orthodontics, Faculty of Dentistry, Ankara Yıldırım Beyazıt University, 06220 Ankara, Türkiye

**Keywords:** cleft lip and palate, orthodontics, maxilla, digital model

## Abstract

**Objective:** To evaluate the measurements made on digital scans of maxillary plaster models in comparison with those obtained directly with a digital caliper on plaster models obtained from individuals with unilateral cleft lip and palate. **Methods:** This study included 42 unilateral cleft lip and palate cases and a control group of 43 Angle Class I cases. The research material consisted of maxillary orthodontic plaster models obtained from these individuals and three-dimensional digital models obtained by scanning these models with a 3 Shape Trios scanner. A total of 12 anatomic reference points were used and six transverse dimension parameters were measured. The differences between the two groups were examined with a Student’s *t*-test. Intraclass correlation coefficients were calculated for repeatability and similarity evaluations. **Results:** Significant differences were found between the CLP and control groups for all parameters, with smaller values obtained in the CLP group. In the CLP group, when comparing the asymmetry of the right and left regions in the 3 Shape model, significant differences were observed regarding all parameters (*p* < 0.05); furthermore, there was a significant difference between the CLP and control groups (*p* < 0.05) in the asymmetry comparison. In both groups, there was no statistically significant difference in the measured parameters between the 3 Shape and digital caliper measurements. **Conclusions:** The measurements obtained after scanning plaster models from CLP individuals with the 3 Shape digital scanner are acceptable and reliable. It can be concluded that the transfer of CLP patients’ archived plaster models to the digital environment is reliable regarding scientific research and clinical measurements.

## 1. Introduction

Cleft lip and palate are among the most common congenital malformations in the craniofacial region, with their incidence varying depending on factors such as geography, ethnicity, and gender [1,2].

It has been reported that 70% of unilateral cleft palates are on the left side. Unilateral clefts in boys are mostly on the left side while, in girls, they are typically on the right side. It has also been reported that the incidence of unilateral primary and secondary cleft palate is approximately three times higher than bilateral cleft palate [1,2]. Dental arch development in individuals with cleft lip and palate is different from that in individuals with normal growth and development patterns [3]. Differences in maxillary arch dimensions and maxillary arch form depend on the cleft type, the amount of tissue present in the cleft area, the relationships between alveolar segments, the individual’s growth and development potential, and individual anatomical characteristics.

Many researchers are of the opinion that the scar tissue formed because of surgical interventions in individuals with cleft lip and palate negatively affects craniofacial growth and development [4,5]. It is thought that lip operations reduce the total arch length and inter-canine width, while palate operations reduce the width between the molars.

Scar tissue may cause a decrease in the anterior alveolar width and, thus, narrowing of the maxillary arch [6]. Lip and palate operations and the resulting scar tissue and abnormal muscle forces negatively affect the growth and development of the upper jaw and the size and shape of the upper tooth arch in individuals with primary and secondary cleft palate. Since the area most affected by this situation is related to the horizontal width of the upper jaw, there have been many studies on this subject [6,7,8,9,10,11].

Examining previous studies evaluating the maxillary and mandibular arch forms of cases with unilateral and bilateral primary and secondary cleft palate, some have focused on evaluating the arch form; for example, the amount of collapse in the maxillary segments and the contact relationships between the maxillary segments have been examined [9,12,13]. Some researchers have evaluated the arch form by examining occlusal relationships [14,15].

It has been stated that in individuals with unilateral primary and secondary cleft palate, the maxillary segments come closer to each other and the cleft width decreases after the lip operation, but collapse occurs in the maxillary segments, especially in the lateral segment on the cleft side [16]. In cases with unilateral cleft lip and palate, the effectiveness of digital scanners and manual methods practically applied in the clinic for transversal direction measurements of the maxillary arch is very important.

Based on this, the aim of this study was to examine the reliability of measurements made on maxillary digital models obtained using a digital scanner in individuals with unilateral cleft lip and palate in comparison with measurements obtained directly with a digital caliper on plaster models, which are accepted as the ‘gold standard’.

## 2. Materials and Methods

The study was approved by the Research Ethics Committee of Ankara University Faculty of Dentistry (Meeting number/Decision date: 36290600/39/21 April 2016). This study included 42 unilateral cleft lip and palate (CLP) cases (24 males, 18 females; aged between 12 and 18 years, mean age 14.75 years)—30 left and 12 right—who applied to Ankara University, Faculty of Dentistry, Department of Orthodontics between 2000 and 2017. A control group of 43 Angle Class I cases (24 males, 19 females; mean age 14.32 years) who showed normal growth and development and did not have a cleft lip and palate anomaly were also included.

The research material consists of maxillary orthodontic plaster models obtained from these individuals and three-dimensional digital models obtained by scanning these models with a 3 Shape Trios (TRIOS POD, 3 Shape, Copenhagen, Denmark) intraoral scanner.

Inclusion criteria for the cleft lip and palate group were as follows:

· Not having any kind of syndrome;
· Not having any kind of neurological or mental problem;
· Being in the permanent dentition period;
· Having unilateral cleft lip and palate;
· Not having received orthodontic treatment;
· Not having different ethnic origins.

Inclusion criteria for the control group were as follows:

· Not having any syndrome, craniofacial anomaly, or cleft lip and palate;
· No history of head–face trauma or any surgical operation in the facial area;
· Not having any kind of neurologic or mental problem;
· Having Angle Class I canine and molar relationships;
· Being in the permanent dentition period;
· Not having received orthodontic treatment;
· Having a similar chronological age to the CLP group;
· Having a similar gender distribution as the CLP group;
· Not having different ethnic origins and racial characteristics.

### 2.1. Study Design

#### 2.1.1. Preparation of Orthodontic Models

The orthodontic models were prepared using alginate impression material (Zhermack, Polesine Badia, Italy) and plastic impression trays. Orthodontic models were obtained from the impressions with type IV hard plaster. Maxillary dental arch models that were directly affected by the cleft lip and palate were used in this study.

#### 2.1.2. Transferring Orthodontic Models to 3D Digital Models and Performing Applications

**Figure 1:**
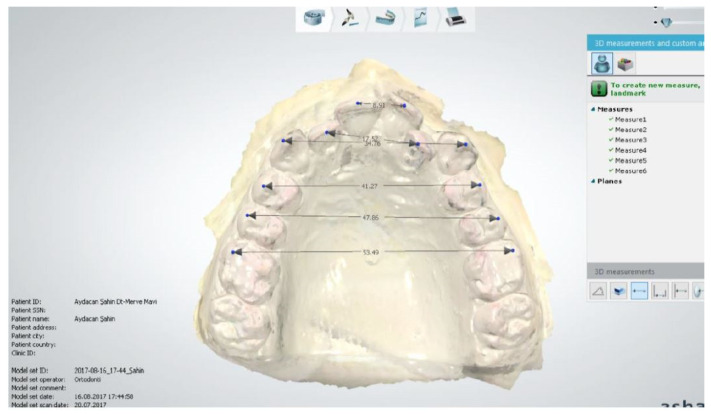
Measurements in the 3 Shape Ortho Analyzer program.

Three-dimensional digital models were obtained by scanning the maxillary orthodontic models prepared based on measurements taken from individuals in both groups with a 3 Shape Trios (TRIOS POD, 3 Shape, Copenhagen, Denmark) intraoral scanner. The obtained data were transferred to the 3 Shape Ortho Analyzer (Copenhagen, Denmark) program and digital measurements were made in this program (Figure 1).

**Figure 2:**
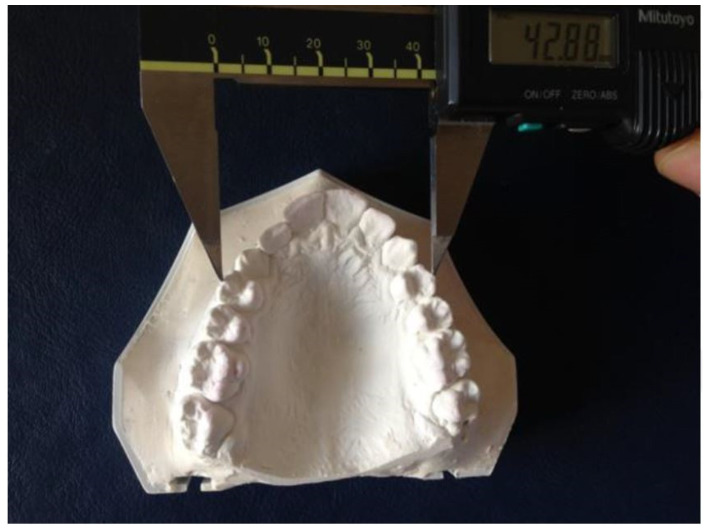
Measurement using digital caliper directly on orthodontic plaster models.

The same measurements were also made using a digital caliper (Mitutoyo Corp., Tokyo, Japan) on our orthodontic plaster study models (Figure 2).

All data for the CLP and control groups were compared for all parameters examined. At the same time, within-group comparisons of the CLP and control groups were undertaken regarding the consistency of the digital caliper and Ortho Analyzer (Copenhagen, Denmark) digital model measurement methods for all parameters.

#### 2.1.3. Anatomical Reference Points (Figure 3)

UR1: Midpoint of the incisive edge of the right central incisor;

UR2: Midpoint of the incisive edge of the right lateral incisor;

UR3: Midpoint of the cusp of the right canine;

UR4: Midpoint of the buccal cusp of the right first premolar;

UR5: Midpoint of the buccal cusp of the right second premolar;

UR6: Midpoint of the mesiobuccal cusp of the right first molar;

UL1: Midpoint of the incisive edge of the left central incisor;

UL2: Midpoint of the incisive edge of the left lateral incisor;

UL3: Midpoint of the cusp of the left canine;

UL4: Midpoint of the buccal cusp of the left first premolar;

UL5: Midpoint of the buccal cusp of the left second premolar;

UL6: Midpoint of the mesiobuccal cusp of the left first molar.

#### 2.1.4. Reference Planes

**Figure 4:**
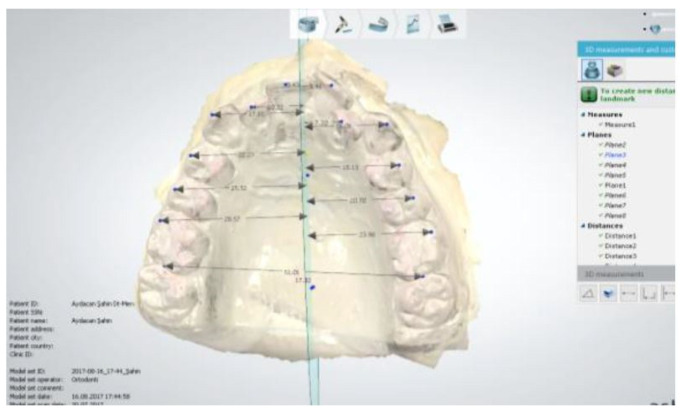
Establishing the midline reference plane.

MRP: Midline reference plane (Figure 4). This is the plane formed by the combination of the midpoint of the right first medial ruga and the left first medial ruga, the midpoint of the right second medial ruga and the left second medial ruga, and the midpoint of the line drawn from the distal contact point of the right first molar to the distal contact point of the left first molar.

#### 2.1.5. Transversal Dimension Parameters

UR1-UL1: Distance between right and left central incisors;

UR2-UR3: Distance between right and left lateral incisors;

UR3-UL3: Distance between right and left canines;

UR4-UL4: Distance between right and left first premolars;

UR5-UL5: Distance between right and left second premolars;

UR6-UL6: Distance between right and left first molars.

### 2.2. Statistical Analysis

In this study, the obtained data were analyzed using the SPSS 21 program (IBM SPSS Statistics for Windows, Version 21.0. Armonk, NY, USA). As a result of the normality tests of the variables, the differences between the two groups were examined with Student’s *t*-test. ICC (Intra Class Correlation Coefficient) reliability coefficients were used for repeatability and similarity evaluations. The significance level was set at 0.05; namely, if *p* < 0.05, it was considered that there was a significant difference, while, if *p* > 0.05, there was no significant difference.

### 2.3. Method Error

To test the reliability of the measurements of all parameters, the measurements for a total of 10 individuals were repeated by the same researcher at least four weeks after the first measurements. The ICC values for the measurements were obtained and all measurements were found to be reliable (ICC 0.953-1).

## 3. Results

### 3.1. Intra-Observer Reliability

Considering the intra-observer reliability in the present study, a high degree of correlation was observed in the repeated measurements for all methods.

### 3.2. Comparison of Two Methods

In the CLP group, when the asymmetry comparison of the right and left regions measured with 3 Shape was examined, a significant difference was observed regarding all parameters (*p* < 0.05).

The UL1-OHD, UL4-OHD, and UL5-OHD values were significantly greater than the UR1-OHD, UR4-OHD, and UR5-OHD values, while the UR2-OHD, UR3-OHD, and UR6-OHD values were significantly greater than the UL2-OHD, UL3-OHD, and UL6-OHD values (Table 1).

In the control group, there was no significant difference in right–left asymmetry in the measurements made with 3 Shape (Table 2).

In the asymmetry comparison, there was a significant difference between the CLP and control groups regarding the values measured with 3 Shape (*p* < 0.05). Only the U1-OHD (left) measurement was found to be significantly higher in the CLP group compared with the control group, while the control group measurements for all other parameters were found to be significantly larger than those in the CLP group (Table 3).

In the CLP group, there was no statistically significant difference in the measured parameters between 3 Shape and digital caliper measurements except for the mean UR1-UL1 measurement, which was 9.31 mm with 3 Shape and 8.69 mm with caliper measurement (Table 4).

In the control group, there was no statistically significant difference in the measured parameters between 3 Shape and digital caliper measurements except for the UR1-UL1 measurement, which was 8.70 mm on average with 3 Shape and 9.31 mm with caliper measurement (Table 5).

ICCs (intraclass correlation coefficients) were calculated for the comparison of 3 Shape and caliper measurement values in the CLP and control groups, and it was found that the measurements were similar (ICC values above 0.75 indicate that the measurement values are similar) (Table 6).

## 4. Discussion

ICCs were calculated to test the reliability of the measurements, and all measurements were found to be reliable (Table 6).

Plaster models are still routinely used due to their advantages such as replicability as a routine technique, low cost, and the possibility to re-evaluate the case later with wax closure [17,18]; however, the disadvantages of plaster models include easy breakage, a margin of error due to abrasion with continuous measurements, and difficulty in archiving due to spatial requirements. Considering these disadvantages, models—which are now accepted as an integral part of orthodontic records—have started to be computerized [18].

Digital models can be created with the support of various software packages and are now used more frequently as records that inform the diagnosis and treatment plan, the effects of treatment, and possible tooth movements. Storing patient information and orthodontic models electronically helps to eliminate problems related to the storage, breakage, reproduction, and maintenance of models; facilitate clinical management and communication between different specialties; and eliminate the abovementioned disadvantages. However, a disadvantage of digital models is the constant dependence on technical support for software.

Whether digital models can replace traditional plaster models has been the subject of various studies [19,20,21,22,23,24]. In previous studies, study groups without DDH anomalies were generally formed and it was reported that the measurements made directly on plaster models were similar to those with the digital 3D scanning method [25].

There are few studies in the literature investigating the reliability of using digital models in individuals with cleft lip and palate deformity, who generally require treatment for many years from birth [26,27,28]. Therefore, our study aimed to evaluate the reliability of measurements made on the digital models of individuals with cleft lip and palate obtained using a digital scanning device in comparison with those for a control group, thus investigating the reliability of digitalizing archived plaster models.

It has been reported that direct intraoral digital scanning in individuals with a cleft lip and palate is preferred by the patient and their guardians, and the comfort of scanning (84.8%) is much higher than that of impression trays (44.2%) [27]. Especially in patients with cleft lip and palate anomalies, classical impression procedures negatively affect the comfort of the patient and pose difficulties for the physician and their assistants due to factors such as insufficient maxilla in the vertical, sagittal, and horizontal directions; insufficient vestibular sulcus depth; and the presence of oronasal fistulas even if the patient is operated. Digital intraoral scans can also eliminate the triggering of the gag reflex in the soft palate. It has also been stated that nasoalveolar shaping performed with the digital modeling method can be considered as an alternative to the classical method [29,30,31].

In the study of Gary et al. comparing different scanners, 3 Shape TRIOS obtained the most successful results regarding accuracy and sensitivity [32]. In another study, accuracy and sensitivity were compared when scanning the entire dental arch using iTero (Align Technology) and Trios (3 Shape) intraoral scanners. They reported that the 3 Shape scanning device—which operates with a newer and more technologically advanced scanning principle—performed better than the old system regarding sensitivity and accuracy, thus providing more clinically acceptable results [19]. We chose 3 Shape as the digital scanning device used in our study because, as stated above, its accuracy, sensitivity, and repeatability were found to be high when compared with other scanning devices.

In the current study, all parameters evaluated in the CLP group were found to be smaller than those in the control group for both measurement methods. It can be speculated that the reason for this situation is the inability of the maxilla to develop sufficiently in the three spatial directions due to the cleft lip and palate deformity and the scar tissues formed due to operations, with corresponding insufficient development of the maxillary dental arch. Studies in the literature have reported similar results [33,34,35,36].

While there were significant differences in the comparison of asymmetry between the right and left regions of the maxillary arch in the cleft lip and palate group, the lack of a statistically significant difference in the control group is an expected outcome, with similar results having been reported in the literature [37,38].

When comparing the asymmetry in the cleft lip and palate group, the distances from the right and left teeth to the midline plane were significantly smaller compared with those in the control group, with only UL1-MRP higher in the CLP group (7.45 mm) than the control group (5.30 mm). This situation can be explained by the fact that 30 of the 42 patients with CLP had a cleft deformity on the left side and the midline was deviated to the cleft side due to the lack of tooth development in this region. Other factors that can cause asymmetries are frequent rotations of the teeth in the deformity area, congenital missing lateral and premolar teeth, or shape and volume anomalies in individuals with CLP deformity [39,40,41].

When comparing the two measurement methods in the CLP and control groups using Student’s *t*-test, all parameters were found to be similar, with only the difference between the right and left central incisors being statistically meaningful. The high anterior crowding in both the control and CLP group models used in our research may have led the measurements made with the caliper to not be as sensitive as those made with the digital models. At the same time, although the difference was found to be statistically significant for both groups, it remained within clinically acceptable limits [42]. When the same comparison was made through ICC testing, it was concluded that the two measurement methods are compatible with each other according to this statistical method, as ICC values above 0.75 were obtained for all measured parameters. Thus, the digital scanning method can be particularly useful in transferring orthodontic patient archives, where traditional plaster models are stored, to a digital platform.

Asquith and McIntyre investigated whether digital models could replace plaster models by comparing both types of models obtained from individuals with unilateral cleft lip and palate anomaly, and reported that 3D digital models can be used as an alternative to traditional plaster models in individuals with unilateral cleft lip and palate deformity [28].

Digital models may prevent the risk of aspiration and respiratory disorders when using impression materials for preoperative jaw treatment of newborns and infants. Okazaki et al. compared the results of both impression methods in the same patient and found that a shift to the 3D printer model is a safe alternative for preoperative jaw correction, as evidenced by the amount of tissue displaced due to the pressure applied during impression-taking [43,44]. 

This study adds new knowledge, as the study sample size was greater than those in previous studies, and a control group was also included. However, this was a retrospective study and the storage conditions of the plaster models may have affected the accuracy. The fact that only one scanner was tested is also a limitation. A larger number of individuals, performing both maxillary and mandibular measurements, and making comparisons according to the severity of the CLP anomaly can be considered among the limitations of this study, serving as a reference for future research.

Reliable digital models positively impact clinical cleft palate care workflows; for example, they enable more accurate surgical planning, facilitate interdisciplinary communication, and are highly beneficial for archiving. While plaster casts of long-term follow-up cases, such as cleft palate cases, require physical storage, reliable digital models eliminate the need for this additional space.

## 5. Conclusions

* For all parameters, smaller values were obtained in the CLP group compared with the control group in both measurement modalities.
* A statistically significant difference was found in the comparison of right–left asymmetry in the CLP group.
* Statistically significant differences were found between the CLP and control groups for all parameters.
* When comparing the two measurement modalities with Student’s *t*-tests in the CLP and control groups, all parameters were found to be similar and only the difference between the right and left central incisors was found to be statistically significant. Although this difference was found to be statistically significant for both groups, it remained within clinically acceptable limits (0.60–0.61 mm).
* The ICCs were found to be high for values obtained with both measurement methods in the CLP and control groups; therefore, both measurement modalities can be considered reliable.

## Figures and Tables

**Figure 3:**
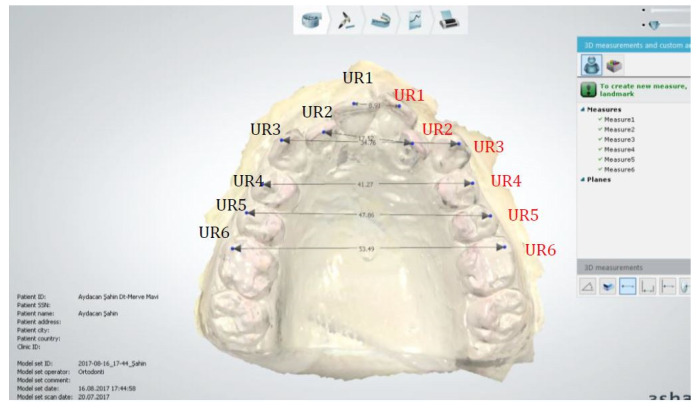
Anatomical reference points.

**Table 1:** Comparison of right–left asymmetry in the CLP group.

		CLP Group						*t*-Test	
		n	Mean	Median	Minimum	Maximum	ss	*t*	*p*
U1-MRP	Right	42	4.16	3.34	1.31	14.69	2.87	−5.4	0.0001
	Left	42	7.45	8.25	1.07	10.59	2.69		
U2-MRP	Right	22	3.21	0.00	0.00	12.39	4.58	2.09	0.043
	Left	22	1.08	0.00	0.00	3.38	1.40		
U3-MRP	Right	42	12.80	13.36	5.24	20.01	4.95	2.24	0.028
	Left	42	10.21	7.53	3.09	19.82	5.62		
U4-MRP	Right	42	12.22	11.00	0.00	25.24	6.56	−2.2	0.031
	Left	42	15.59	16.85	0.00	29.01	7.43		
U5-MRP	Right	42	11.17	10.40	0.00	26.96	5.88	−6.5	0.0001
	Left	42	21.49	24.89	0.00	29.76	8.44		
U6-MRP	Right	42	21.76	20.31	10.49	29.95	6.62	2.29	0.024
	Left	42	18.30	17.06	3.60	29.90	7.19		

**Table 2:** Comparison of right–left asymmetry in the control group.

		Control Group						*t*-Test	
		n	Mean	Median	Minimum	Maximum	sd	*t*	*p*
U1-MRP	Right	43	5.31	5.05	3.64	7.95	1.01	0.04	0.968
	Left	43	5.30	5.21	3.98	7.21	0.92		
U2-MRP	Right	43	12.60	12.24	10.37	15.86	1.38	1.14	0.256
	Left	43	12.28	12.16	10.27	15.89	1.21		
U3-MRP	Right	43	17.69	17.81	15.06	22.37	1.36	0.5	0.617
	Left	43	17.54	17.46	15.23	22.34	1.33		
U4-MRP	Right	43	22.29	22.08	19.37	26.33	1.32	−0.2	0.838
	Left	43	22.35	22.12	19.09	26.76	1.34		
U5-MRP	Right	43	25.39	25.46	22.09	29.06	1.41	−0.03	0.971
	Left	43	25.40	25.39	22.47	29.09	1.35		
U6-MRP	Right	43	27.17	26.88	23.14	30.59	1.67	−0.09	0.924
	Left	43	27.20	27.12	23.17	30.89	1.67		

**Table 3:** Asymmetry comparison between CLP and control groups.

		CLP Group						*t*-Test	
		n	Mean	Median	Minimum	Maximum	sd	*t*	*p*
U1-MRP (right)	CLP	42	4.16	3.34	1.31	14.69	2.87	−2.7	0.015
	Control	43	5.31	5.05	3.64	7.95	1.01		
U1-MRP (left)	CLP	42	7.45	8.25	1.07	10.59	2.69	4.9	0.0001
	Control	43	5.30	5.21	3.98	7.21	0.92		
U2-MRP (right)	CLP	22	3.21	0.00	0.00	12.39	4.58	−12.4	0.0001
	Control	43	12.60	12.24	10.37	15.86	1.38		
U2-MRP (left)	CLP	22	1.08	0.00	0.00	3.38	1.40	−33.4	0.0001
	Control	43	12.28	12.16	10.27	15.89	1.21		
U3-MRP (right)	CLP	42	12.80	13.36	5.24	20.01	4.95	−6.2	0.0001
	Control	43	17.69	17.81	15.06	22.37	1.36		
U3-MRP (left)	CLP	42	10.21	7.53	3.09	19.82	5.62	−8.3	0.0001
	Control	43	17.54	17.46	15.23	22.34	1.33		
U4-MRP (right)	CLP	42	12.22	11.00	0.00	25.24	6.56	−9.8	0.0001
	Control	43	22.29	22.08	19.37	26.33	1.32		
U4-MRP (left)	CLP	42	15.59	16.85	0.00	29.01	7.43	−5.8	0.0001
	Control	43	22.35	22.12	19.09	26.76	1.34		
U5-MRP (right)	CLP	42	11.17	10.40	0.00	26.96	5.88	−15.4	0.0001
	Control	43	25.39	25.46	22.09	29.06	1.41		
U5-MRP (left)	CLP	42	21.49	24.89	0.00	29.76	8.44	−3.1	0.004
	Control	43	25.40	25.39	22.47	29.09	1.35		
U6-MRP (right)	CLP	42	21.76	20.31	10.49	29.95	6.62	−5.1	0.0001
	Control	43	27.17	26.88	23.14	30.59	1.67		
U6-MRP (left)	CLP	42	18.30	17.06	3.60	29.90	7.19	−7.9	0.0001
	Control	43	27.20	27.12	23.17	30.89	1.67		

**Table 4:** Comparison of measurement methods in the CLP group.

		CLP Group					*t*-Test		
		n	Mean	Median	Minimum	Maximum	sd	*t*	*p*
UR1- MRP	3Shape	42	4.18	4.30	2.18	6.55	1.00	0.065	0.948
	Caliper	42	4.17	4.28	2.15	6.53	1.00		
UR2- MRP	3Shape	42	9.37	10.15	2.54	11.76	2.33	0.024	0.981
	Caliper	42	9.35	10.13	2.50	11.75	2.34		
UR3- MRP	3Shape	42	12.17	12.33	3.64	19.01	3.48	0.008	0.994
	Caliper	42	12.17	12.32	3.64	19.03	3.48		
UR4- MRP	3Shape	42	17.04	17.57	7.37	22.98	3.04	0.018	0.986
	Caliper	42	17.03	17.55	7.37	22.95	3.04		
UR5- MRP	3Shape	42	20.12	20.61	11.44	27.83	3.80	0.018	0.985
	Caliper	42	20.10	20.59	11.45	27.85	3.81		
UR6- MRP	3Shape	42	23.62	23.94	16.79	27.58	2.51	0.023	0.981
	Caliper	42	23.61	23.94	16.78	27.59	2.51		
UL1- MRP	3Shape	42	4.33	4.32	1.23	7.33	1.58	0.041	0.967
	Caliper	42	4.31	4.30	1.21	7.29	1.58		
UL2- MRP	3Shape	42	9.09	9.08	2.33	12.51	2.53	0.011	0.992
	Caliper	42	9.08	9.07	2.30	12.49	2.53		
UL3- MRP	3Shape	42	13.28	13.00	5.40	19.70	3.65	0.013	0.991
	Caliper	42	13.27	13.00	5.36	19.68	3.65		
UL4- MRP	3Shape	42	18.39	18.46	11.47	25.93	3.19	0.026	0.981
	Caliper	42	18.37	18.45	11.45	25.94	3.19		
UL5- MRP	3Shape	42	20.78	21.39	10.63	29.52	3.83	0.025	0.981
	Caliper	42	20.76	21.37	10.61	29.51	3.84		
UL6-MRP	3Shape	42	22.41	22.88	15.81	29.90	3.28	0.049	0.961
	Caliper	42	22.38	22.86	15.80	29.60	3.30		
UR1-UL1	3Shape	42	9.31	9.12	5.30	12.20	1.61	2.15	0.034
	Caliper	42	8.69	8.88	5.97	10.99	0.96		
UR3-UL3	3Shape	42	26.11	25.89	16.14	37.23	5.41	0.236	0.814
	Caliper	42	25.83	25.60	16.14	35.43	5.16		
UR4-UL4	3Shape	42	36.71	35.95	28.64	47.29	4.32	0.305	0.761
	Caliper	42	36.42	35.84	26.27	47.25	4.57		
UR5-UL5	3Shape	42	41.65	42.55	21.03	51.86	7.00	0.017	0.986
	Caliper	42	41.63	42.51	21.01	51.82	6.99		
UR6-UL6	3Shape	42	49.06	49.98	33.62	55.72	4.06	1.26	0.211
	Caliper	42	46.89	49.74	1.03	55.69	10.41		

**Table 5:** Comparison of measurement methods in the control group.

		Control Group						*t*-Test	
		n	Mean	Median	Minimum	Maximum	sd	*t*	*p*
UR1-MRP	3Shape	43	5.26	5.03	3.64	7.95	1.04	0.012	0.991
	Caliper	43	5.26	5.05	3.65	7.94	1.04		
UR2-MRP	3Shape	43	12.34	12.11	10.14	15.86	1.50	0.049	0.961
	Caliper	43	12.33	12.08	10.12	15.86	1.51		
UR3-MRP	3Shape	43	17.55	17.69	15.53	21.37	1.14	0.057	0.955
	Caliper	43	17.53	17.54	15.56	21.37	1.14		
UR4-MRP	3Shape	43	21.22	21.08	16.64	24.33	1.45	0.047	0.962
	Caliper	43	21.20	21.06	16.62	24.31	1.45		
UR5-MRP	3Shape	43	24.17	24.20	21.22	28.01	1.36	0.053	0.958
	Caliper	43	24.15	24.18	21.20	28.02	1.37		
UR6-MRP	3Shape	43	26.62	26.54	22.70	30.59	1.74	0.032	0.975
	Caliper	43	26.61	26.51	22.69	30.58	1.74		
UL1-MRP	3Shape	43	4.75	5.02	3.04	6.09	0.79	0.067	0.947
	Caliper	43	4.74	5.00	3.03	6.07	0.79		
UL2-MRP	3Shape	43	11.65	11.56	10.12	13.67	0.97	0.071	0.944
	Caliper	43	11.63	11.57	10.00	13.65	0.98		
UL3-MRP	3Shape	43	18.19	18.09	14.67	22.34	1.47	0.039	0.969
	Caliper	43	18.18	18.08	14.66	22.33	1.47		
UL4-MRP	3Shape	43	22.29	22.09	19.45	26.76	1.39	0.051	0.959
	Caliper	43	22.28	22.07	19.43	26.75	1.39		
UL5-MRP	3Shape	43	25.62	25.52	22.67	29.09	1.31	0.04	0.968
	Caliper	43	25.61	25.51	22.64	29.06	1.32		
UL6-MRP	3Shape	43	26.48	26.78	22.14	30.76	1.99	0.034	0.973
	Caliper	43	26.46	26.77	22.13	30.75	1.99		
UR1-UL1	3Shape	43	8.70	8.90	5.97	10.99	0.96	−2.14	0.034
	Caliper	43	9.31	9.19	5.30	12.20	1.59		
UR3-UL3	3Shape	43	34.87	35.38	31.58	39.04	1.94	−0.002	0.997
	Caliper	43	34.87	35.35	31.55	39.00	1.95		
UR4-UL4	3Shape	43	41.99	42.57	34.23	45.98	2.73	−0.07	0.944
	Caliper	43	42.04	42.79	34.20	45.95	2.78		
UR5-UL5	3Shape	43	47.63	48.05	39.38	52.09	2.63	0.086	0.932
	Caliper	43	47.59	48.01	39.35	52.05	2.65		
UR6-UL6	3Shape	43	50.55	51.82	36.50	56.55	4.40	0.033	0.994
	Caliper	43	50.52	51.81	36.48	56.52	4.40		

**Table 6:** Intraclass correlations between measurement methods in the CLP and control groups.

ICC (Intraclass Correlation Coefficient)		ICC
UR1-MRP	CLP	1
	Control	1
UR2-MRP	CLP	1
	Control	1
UR3-MRP	CLP	1
	Control	0.999
UR4-MRP	CLP	1
	Control	1
UR5-MRP	CLP	1
	Control	1
UR6-MRP	CLP	1
	Control	1
UL1-MRP	CLP	1
	Control	1
UL2-MRP	CLP	1
	Control	1
UL3-MRP	CLP	1
	Control	1
UL4-MRP	CLP	1
	Control	1
UL5-MRP	CLP	1
	Control	1
UL6-MRP	CLP	0.999
	Control	1
UR1-UL1	CLP	0.995
	Control	0.999
UR3-UL3	CLP	0.82
	Control	0.999
UR4-UL4	CLP	0.967
	Control	0.998
UR5-UL5	CLP	1
	Control	0.991
UR6-UL6	CLP	1
	Control	0.991

## Data Availability

The data presented in this study are available upon request from the corresponding authors.
